# Supervisor psychological closeness and employees’ taking charge at work: a moderated mediated model based on trust in supervisor and employee power distance orientation

**DOI:** 10.3389/fpsyg.2026.1731160

**Published:** 2026-04-29

**Authors:** Xiaohua Feng, Shagufta Zada, Hammad S. Alotaibi, Muhammad Zada, Kishwar Ali, Jawad Khan

**Affiliations:** 1 School of Foreign Language, Hanjiang Normal University, Shiyan, China; 2 College of Taraba, Taif University, Taif, Saudi Arabia; 3 Facultad de Administración y Negocios, Universidad Autónoma de Chile, Santiago, Chile; 4 Applied Science Research Center, Applied Science Private University, Amman, Jordan; 5 Advanced Research Centre, European University of Lefke, Lefke, Northern Cyprus, Türkiye; 6 ARUCAD Research Centre, Arkin University of Creative Arts and Design, Northern Cyprus, Türkiye

**Keywords:** employee power distance orientation, employees’ taking charge at work, social exchange theory, supervisor psychological closeness, trust in supervisor

## Abstract

**Purpose:**

Drawing on social exchange theory, this study investigates the relationship between supervisors’ psychological closeness and employees’ taking charge at work. Furthermore, it examines the mediating role of trust in the supervisor and the moderating effect of employee power distance orientation.

**Design/methodology/approach:**

The study employs structural equation modeling in Mplus (version 8.6) to analyze multi-source data collected from a diverse sample of employees and supervisors, testing the proposed relationships.

**Findings:**

The findings indicate that supervisors’ psychological closeness positively impacts employees’ taking charge at work. Trust in the supervisor mediates this relationship, providing insights into the underlying psychological mechanisms. Additionally, employee power distance orientation moderates the link between psychological closeness and trust in the supervisor, thereby influencing the indirect effect of psychological closeness on taking-charge behaviors through trust.

**Originality/value:**

This study offers a deeper understanding of how relational and cultural factors, including supervisor closeness and power distance orientation, jointly influence proactive work behaviors. It contributes to the literature by highlighting the relationship between psychological and cultural factors in shaping employees’ work behaviors.

## Introduction

In the face of today’s increasingly volatile and evolving work landscape, the success and longevity of organizations depend more than ever on the proactive behaviors of their employees—those that are initiated by the individual, oriented toward change, and focused on long-term goals (Ngo et al., 2023). Among the various forms of proactive behavior, *employees taking charge at work*—defined as voluntary and constructive efforts by employees to bring about functional change in how work is executed within their roles or teams (Zhang et al., 2021) — has gained considerable scholarly and managerial attention. This behavior is especially valuable in dynamic work environments that demand continuous adaptation and innovation (Liu et al., 2021).

To date, researchers have identified several individual, relational, and contextual antecedents that facilitate taking charge behavior. For instance, individual-level factors such as proactive personality (Guzman et al., 2024), intrinsic motivation (Kim S. S. et al., 2023), and psychological empowerment (Kumar et al., 2022) have been shown to positively influence employees’ likelihood of taking charge. At the relational level, leadership style has emerged as a key driver—transformational leadership (Du and Yan, 2022), ethical leadership (Wang et al., 2020), and servant leadership (Xu et al., 2022) have all been linked to higher levels of taking charge. In terms of contextual factors, a supportive organizational climate and job autonomy have also been found to foster an environment in which proactive behaviors can flourish (Liu Y. et al., 2021).

However, despite the recognized importance of taking charge, much remains unknown about the social and psychological mechanisms that cultivate such behavior (Zhang et al., 2021). In particular, the quality of the supervisor-employee relationship appears to be a critical but underexplored determinant of employee proactivity (Kim S. S. et al., 2023). Although social exchange theory (SET) (Blau, 1964) has provided the foundation for understanding dyadic relationships at work, finer-grained relational constructs such as supervisors’ psychological closeness remain under-investigated in the context of proactive work behaviors (Liborius et al., 2025).

Central to the social fabric of any organization is the dyadic relationship between supervisors and their subordinates (Kim et al., 2023b). Supervisors play a pivotal role in shaping employee behavior, attitudes, and perceptions through daily interactions and the emotional climate they foster. One such influential element *is* supervisors’ psychological closeness, which refers to the degree of emotional intimacy, accessibility, and interpersonal warmth that supervisors display towards their subordinates (Graen and Uhl-Bien, 1995). Psychological closeness can foster a supportive and valued environment for employees (Gino and Galinsky, 2012), encouraging them to take risks and engage in proactive behaviors, such as stepping up and taking charge (Cangiano et al., 2021). Building on social exchange (Blau, 1964), we argue that psychological closeness from supervisors is not merely a relational attribute but a potent catalyst for eliciting discretionary effort from employees. Building on this idea, we contend that employees who feel psychologically close to their supervisors are more likely to feel comfortable expressing ideas and taking proactive actions (Love and Dustin, 2014).

However, the link between psychological closeness and taking charge may not be direct. We propose that trust in a supervisor acts as a mediating mechanism that channels the influence of psychological closeness into taking charge at work. Psychological closeness captures the extent to which employees feel a sense of relational connection, mutual understanding, and interpersonal overlap with their supervisors. Such closeness provides the relational context from which trust can emerge. Although trust and psychological closeness are related, they are conceptually distinct. Psychological closeness reflects a felt sense of interpersonal connection, whereas trust reflects a willingness to accept vulnerability based on positive expectations of the supervisor’s intentions and behavior (Li and Tan, 2013). In other words, feeling close to a supervisor does not automatically mean that an employee will act proactively; rather, this relational closeness first helps employees develop trust that the supervisor will respond fairly, supportively, and without punitive consequences. Thus, trust is positioned as a mediating mechanism through which psychological closeness is translated into taking charge at work.

This sequence is consistent with social exchange theory (Blau, 1964), which suggests that repeated positive relational experiences create the foundation for trust-based exchanges. When employees experience psychological closeness with their supervisors, they are more likely to interpret the relationship as supportive, respectful, and dependable (Knoll and Gill, 2011). These relational cues strengthen trust in the supervisor, which then reduces the interpersonal risk associated with taking charge. Because taking charge involves challenging existing practices, suggesting change, and stepping beyond formal job requirements, employees are unlikely to engage in such behavior unless they believe their supervisors will respond favorably rather than defensively (Basit, 2021). Trust therefore serves as the more proximal psychological mechanism that converts relational closeness into proactive action. In this way, psychological closeness functions as an antecedent relational condition, whereas trust operates as the motivational and risk-reducing pathway through which employees become willing to take charge at work.

The urgency of clarifying the moderating role of cultural values becomes particularly evident in the era of globalization. Given the growing cultural diversity in organizations, it is essential to acknowledge how cultural values shape employees’ responses to various work-related behaviors. The cross-cultural management literature (Du et al., 2022) suggests that employees’ cultural values are crucial in determining how they perceive and respond to leadership behaviors. To effectively manage employees, it is essential to gain a deeper understanding of how these cultural values influence employees’ interpretations of leaders’ behaviors and how these interactions subsequently affect their psychological and behavioral outcomes (Pervaiz et al., 2021).

In the present study, we specifically investigate the moderating role of individual power distance orientation for two key reasons. First, power distance is one of the most prominent cultural values across numerous cultural frameworks (Matta et al., 2022). Second, power distance is particularly relevant to the current research because it directly affects how employees perceive and respond to supervisor behaviors, especially regarding authority and power dynamics (Wen et al., 2025). Employees’ fundamental values regarding power influence their understanding of, and reactions to, supervisory behaviors, including psychological closeness (Guo et al., 2022; Peltokorpi and Ramaswami, 2021). In particular, employees with a high power distance orientation tend to perceive authority figures as formal, distant, and inherently superior in status (Shahzad et al., 2024). By contrast—and of greater relevance to our findings—employees in our context exhibit a *low* power distance orientation, meaning they are more comfortable with egalitarian relationships and expect supervisors to interact in a personable and approachable manner (Peltokorpi and Ramaswami, 2021). For these employees, supervisor psychological closeness is more likely to be interpreted as a signal of mutual respect, accessibility, and genuine support (Lei and Rau, 2021). Thus, recognizing employees’ low power distance orientation is essential to understanding why relational cues, such as supervisors’ psychological closeness, are readily embraced and positively internalized in this setting (Rubenstein et al., 2025).

**Figure 1:**
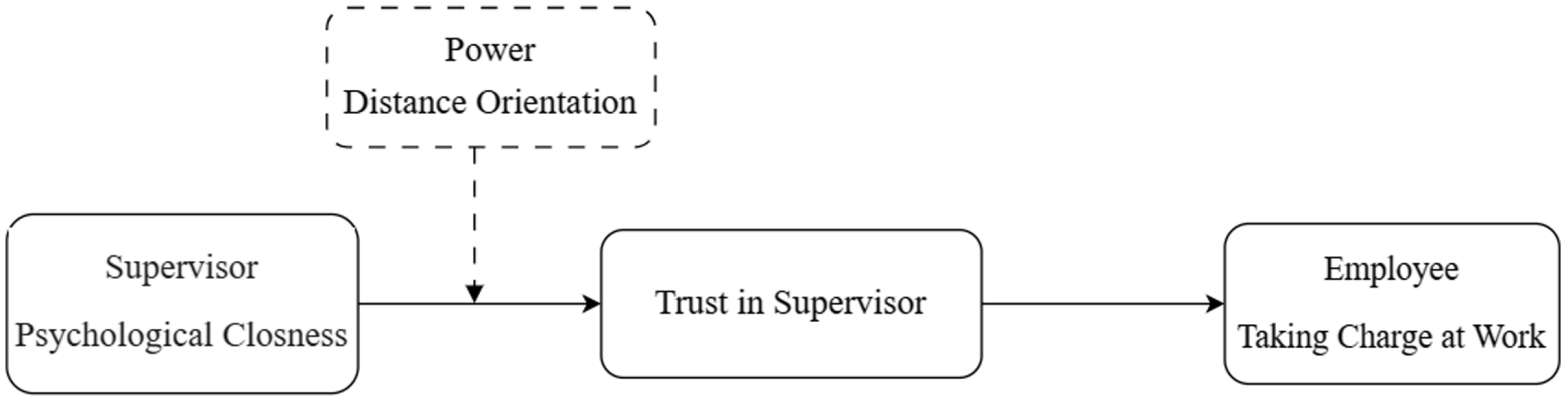
Conceptual model.

This study contributes to the literature in many ways. First, we extend the understanding of the antecedents of taking charge behavior by introducing supervisors’ psychological closeness as a critical, yet underexplored factor. While prior research has predominantly focused on structural or trait-based predictors of proactive behavior, our study shifts the focus toward the relational context of the supervisor-employee dyad. By doing so, we highlight how effective proximity and perceived emotional availability of supervisors can serve as critical psychological resources that energize employees to take initiative. Second, by introducing trust in the supervisor as a mediating mechanism, we offer a more nuanced theoretical explanation of how and why psychological closeness influences proactive outcomes. This contribution is particularly valuable as it uncovers the underlying social-cognitive processes through which high-quality supervisory relationships foster employee agency. Trust, in this sense, acts as a psychological conduit, enabling employees to feel secure, supported, and thus more willing to challenge the status quo through taking charge behavior. Third, we incorporate employee power distance orientation as a boundary condition that shapes the relational dynamic between supervisors and subordinates. This cultural and individual differences perspective allows us to theorize about the contingent nature of relational influences, showing that the impact of psychological closeness is not uniform across employees. Instead, it is filtered through individuals’ culturally informed values and expectations regarding hierarchy and authority. This enriches the literature by integrating a cross-cultural sensitivity into existing models of proactive behavior, acknowledging that managerial efforts may be interpreted and internalized differently across diverse workplace settings. The conceptual model is shown in Figure 1.

## Literature review and hypotheses development

### Supervisor psychological closeness and employee taking charge at work

Supervisor psychological closeness refers to the feelings of attachment and perceived connection between supervisors and employees (Liborius et al., 2025). In organizational settings, when employees perceive psychological closeness with their supervisors, it signals a high-quality social exchange relationship (Berkovich and Eyal, 2018). Drawing on social exchange theory (Blau, 1964), such relationships are sustained through ongoing exchanges of socioemotional resources and a general expectation of reciprocity over time (Akkermans et al., 2024). The norm of reciprocity (Gouldner, 1960) suggests that individuals are motivated to reciprocate when they receive favorable treatment. In such a context, psychological closeness with supervisors may encourage employees to respond with proactive and extra-role behaviors that contribute positively to the organization (Kim S. S. et al., 2023; Lee, 2021).

Taking charge is one such discretionary behavior, defined as employees’ voluntary and constructive efforts to improve how work is performed, how roles are structured, or how organizational processes unfold (Kim et al., 2023a). Unlike routine job performance or conventional forms of citizenship behavior, taking charge is inherently change-oriented, focusing on initiating improvements and driving innovation (Li and Chen, 2019). Supervisor psychological closeness can encourage employees to take charge by creating a relational foundation that empowers them to pursue change initiatives confidently (Hui et al., 2003). When employees feel emotionally connected to their supervisors, characterized by a sense of intimacy, familiarity, and shared identity, they are more likely to feel valued and contribute proactively to organizational development (Wu and Parker, 2017).

*Hypothesis 1*: Supervisor psychological closeness is positively related to employee taking charge at work.

### Trust in supervisor as a mediator

Psychological closeness in leadership refers to the strength and quality of personal connections between leaders and their team members, extending beyond formal roles or organizational hierarchy (Grant et al., 2009). It is rooted in the supervisor’s ability to foster mutual understanding and shared purpose, shaping employees’ perception of their value within the organization (Therkelsen and Fiebich, 2004). Unlike transactional interactions, psychological closeness emphasizes rapport and alignment (Finkel et al., 2017), where supervisors prioritize not only task-related outcomes but also the holistic wellbeing and growth of subordinates (Gino and Galinsky, 2012). Psychological closeness with the supervisor reflects employees’ perceived emotional connection to their supervisor, fostering trust by perceiving the supervisor as reliable, fair, and benevolent (Ariani, 2015).

Social exchange theory posits that organizational relationships are governed by reciprocity (Cropanzano et al., 2017). When supervisors invest in building psychologically close relationships through empathy, transparency, and emotional support, employees feel obliged to reciprocate (e.g., trust in the supervisor) (Li and Tan, 2013). Supervisors who prioritize psychological closeness cultivate high-quality exchanges, distinguishing themselves from transactional leaders (Kim et al., 2016). Trust, once established, becomes the psychological mechanism enabling employees to take charge (Ugwu et al., 2014). Taking charge at work involves voluntary actions aimed at improving work processes, such as suggesting innovations, addressing inefficiencies, or challenging outdated practices (Kim et al., 2023b). Trust in supervisors encourages employees to engage in taking charge behaviors, defined as voluntary efforts to initiate constructive change, such as voicing ideas or addressing inefficiencies, even when interpersonal risks are involved (Basit, 2021). An employee trusting their supervisor is more likely to propose a new workflow strategy, confident that their input will be reasonably considered (Knoll and Gill, 2011).

*Hypothesis 2:* Trust in the supervisor mediates the relationship between supervisor psychological closeness and employee taking charge at work.

### Employee power distance orientations as a moderator

Social exchange theory (Blau, 1964) posits that social behavior is driven by an exchange process aimed at maximizing benefits and minimizing costs. In the workplace, positive interactions between supervisors and employees are seen as exchanges that build mutual trust, obligation, and loyalty over time (Finkel et al., 2017; Love and Dustin, 2014). Supervisor behaviors that convey warmth, personal attention, and psychological closeness serve as essential relational resources, signaling support and respect for employees (Liborius et al., 2025). In response to such resources, employees are motivated to reciprocate with positive attitudes and behaviors, including greater trust in their supervisors and proactive efforts at work (Finkel et al., 2017; Kim S. S. et al., 2023).

However, the quality and intensity of these social exchanges may be influenced by individual values, particularly power distance orientation, which captures the degree to which individuals view unequal power distribution within organizations as acceptable (Hofstede, 1980). Although initially conceptualized at the societal level, scholars have demonstrated meaningful variations in power distance orientation at the individual level (Zhang and Begley, 2011). Following Begley et al. (2002), we treat power distance orientation as an individual characteristic. Because power distance orientation reflects how individuals perceive and react to authority relationships, it should influence how employees interpret and respond to a supervisor’s efforts to build close relationships (Fock et al., 2013).

Employees with stronger power distance orientation tend to regard hierarchical differences as appropriate and are therefore more inclined to defer to supervisory authority. For these employees, psychological closeness from a supervisor may be perceived as less critical to developing trust, because respect and obedience are already granted based on hierarchical position alone (Yang, 2020). However, although hierarchy is important for high-power-distance employees, research suggests that perceptions of fair and consistent treatment still play a meaningful role in determining whether such employees trust their supervisors (Nienaber et al., 2015). When high power distance employees feel that rules, rewards, and sanctions are applied consistently across individuals—regardless of status—they may perceive the supervisor as just, thereby strengthening trust (Colquitt, 2004; Colquitt et al., 2001). Thus, even in high power distance contexts, employees evaluate whether the supervisor’s behavior aligns with principles of equity and moral appropriateness before deepening trust. As such, the positive impact of psychological closeness may remain limited, but perceptions of fair treatment can still reinforce trust, even among employees with high power distance (Li and Chen, 2019).

In contrast, employees with a low power distance orientation value egalitarian relationships and are less inclined to automatically defer to authority (Lin et al., 2013). For them, trust in a supervisor is not granted solely based on position but must be earned through interpersonal warmth, respect, and support (Ji et al., 2015). Low power distance employees are also particularly sensitive to cues of justice and equity because they expect supervisors to treat them as equals. When psychological closeness is accompanied by respectful communication, participative decision-making, and demonstrations of fairness, low-power-distance employees interpret such cues as evidence that the supervisor honors principles of equality and mutual respect. This strengthens perceptions of procedural and interactional justice, thereby deepening trust (Costigan et al., 2006). We therefore argue that when supervisor psychological closeness aligns with employees’ expectations for mutual respect, fairness, and collaboration, it fosters a stronger sense of trust. Thus, social exchange processes are more active and salient for individuals with low power distance when supervisors offer psychological closeness (Li and Cropanzano, 2009).

Trust in the supervisor, in turn, facilitates employees taking charge behaviors—self-initiated, change-oriented actions aimed at improving work processes and environments (Willemyns et al., 2003). When employees trust their supervisors, they are more willing to take interpersonal risks, such as suggesting new ideas or challenging existing practices, because they expect support rather than punishment in response (Yagil, 2014). Thus, supervisor psychological closeness fosters trust, which encourages employees, particularly those with low power distance orientation, to engage in proactive behaviors.

*Hypothesis 3a:* Employee power distance orientation moderates the association between supervisor psychological closeness and employee trust in the supervisor, such that the relationship is stronger for employees who have lower power distance orientation.

*Hypothesis 3b:* Employee power distance orientation indirectly moderates the association between supervisor psychological closeness and employee taking charge at work, via employee trust in the supervisor, such that the relationship is stronger for employees with lower power distance orientation.

## Methods

### Sample and procedure

The data were obtained from employees and their supervisors across 21 companies in Beijing and Shenzhen, China. The participating organizations represented diverse industries, including banking, transportation, electronics, and hospitality. To reduce common method bias, data were collected in three phases, with one-month intervals between waves (Podsakoff et al., 2003). At Time 1, questionnaires were distributed to 170 supervisors and 240 subordinates. Supervisors rated their psychological closeness to the focal employee, while employees reported power distance orientation and demographic information. At Time 2, the same employees were invited to complete a second survey measuring trust in the supervisor. At Time 3, supervisors evaluated employees’ taking charge at work. All questionnaires were pre-coded to ensure accurate matching across the three waves. After excluding incomplete and unmatched responses, the final sample comprised 158 matched supervisor–subordinate dyads. A *post hoc* power analysis based on Cohen’s (1988) guidelines indicated that, assuming a medium effect size (*f*^2^ = 0.15) and *α* = 0.05, the final sample of 158 matched dyads provided adequate statistical power (1 − *β* = 0.99) for the key regression equation. Even under more conservative specifications, the statistical power remained above 0.97, supporting the adequacy of the sample size. On average, respondents were between 30 and 45 years old and had worked with their current supervisor for approximately 5 years. Around 76% held a bachelor’s degree or higher.

### Measures

#### Supervisor psychological closeness

Supervisor psychological closeness was measured using the Inclusion-of-Other-in-the-Self (IOS) Scale (Aron et al., 1992), a validated visual measure commonly used to assess subjective closeness and interpersonal connection. The IOS depicts seven pairs of circles labelled “Self” and “Other” that vary in their degree of overlap (see Appendix A). This pictorial representation serves as the “sample items” of the scale: supervisors choose the diagram that best reflects how close or interconnected they feel with a specific employee. The IOS has demonstrated strong construct validity in both interpersonal and organizational contexts, showing reliable associations with related constructs such as trust, identification, and relational bonding (Gächter et al., 2015; Schubert and Otten, 2002). In this study, supervisors selected one of the seven diagrams, ranging from 1 (no overlap, indicating low psychological closeness) to 7 (almost complete overlap, indicating high psychological closeness). Scores, therefore, directly reflect the supervisor’s perceived level of relational closeness and interconnectedness with the employee. Higher values represent stronger perceived psychological closeness.

#### Trust in supervisor

We used McAllister (1995) operationalization, adding an overall item that was adjusted to say, “I trust and respect my supervisor.” Additionally, we modified McAllister’s scale to refer specifically to the supervisor. Other sample items are “My supervisor approaches his or her job with professionalism and dedication” and “I have a sharing relationship with my supervisor. I can freely share my ideas, feelings, and hopes with him or her.”

### Employee taking charge at work

To measure employees taking charge at work, the supervisor used a 10-item scale developed by Morrison and Phelps (1999). A sample item was “This employee often tries to adopt improved procedures for doing his or her job.”

### Power distance orientation

Drawing on previous individual-level studies (Brockner et al., 2001; Kim and Leung, 2007), we assessed power distance orientation using an eight-item scale developed by Earley and Erez (1997). Sample item is “In work-related matters, managers have a right to expect obedience from their subordinates.”

### Control variables

Consistent with prior research on proactive behavior (i.e., taking charge at work) (Liu D. et al., 2021; Xu et al., 2023; Zeng et al., 2020), we controlled employees ‘gender, age, education level, and tenure with their supervisor.

## Results

### Means and correlations

Table 1 presents the means, standard deviations, and correlations among the study variables. Supervisor psychological closeness was positively correlated with employee trust in supervisor (*r* = 0.43, *p* < 0.01) and employee taking charge at work (*r* = 0.52, *p* < 0.01). Employee trust in supervisor was also positively correlated with employee taking charge at work (*r* = 0.40, *p* < 0.01).

**Table 1:** Means, standard deviations, and correlations among study variables.

Variable	*M*	SD	1	2	3	4
1. Supervisor psychological closeness	3.99	1.73	—			
2. Power distance orientation	3.02	1.02	−0.01	—		
3. Trust in supervisor	3.11	1.03	0.43**	0.15	—	
4. Taking charge at work	3.09	1.01	0.52**	0.06	0.40**	—

**p < 0.01.

### Measurement model

A series of CFA was conducted to examine the measurement model and assess discriminant validity. As reported in Table 2, the hypothesized four-factor model, comprising supervisor psychological closeness, trust in supervisor, employee taking charge at work, and power distance orientation, demonstrated a superior fit to the data relative to the alternative models. Specifically, the four-factor model yielded the following fit indices: χ^2^ = 225.80, df = 160, χ^2^/df = 1.41, RMSEA = 0.05, SRMR = 0.06, CFI = 0.92, and TLI = 0.91. These results indicate an acceptable fit and support the distinctiveness of the study constructs.

**Table 2:** Measurement model.

Models	Factors	χ^2^	df	χ^2^/df	Δχ^2^	RMSEA	SRMR	CFI	TLI
M 1	4 Factors: SPC, ETS, ETCW, EPDO	225.80	160	1.41	----	0.05	0.06	0.92	0.91
M 2	3 Factors: SPC + ETCW, ETS, EPDO	1402.55	162	8.66	1176.75	0.14	0.12	0.75	0.79
M 3	1 Factor: SPC + ETS + ETCW + EPDO	3550.3	166	21.39	2147.75	0.18	0.20	0.49	0.45

SPC = Supervisor Psychological Closeness; ETS = Employee Trust in the Supervisor; ETCW = Employee Taking Charge at Work; EPDO = Employee Power Distance Orientation.

### Hypotheses testing

The hypotheses were tested using SEM in Mplus version 8.6. To examine the indirect and moderated mediation effects, bias-corrected bootstrapping with 5,000 resamples was used to generate 95% confidence intervals. As shown in Table 3, supervisor psychological closeness had a positive effect on employee taking charge at work (*B* = 0.31**, SE = 0.04, 95% CI [23, 38]), supporting Hypothesis 1. Hypothesis 2 proposed that trust in supervisor would mediate the association between supervisor psychological closeness and employee taking charge at work. The results supported this prediction, as the indirect effect was significant (*B* = 0.05**, BootSE = 0.02, 95% CI [0.02, 0.09]).

**Table 3:** Hypotheses results.

Hypotheses	Path’s	*B*	SD	95% CI	Decision
Hypothesis 1	Supervisor psychological closeness → Employee taking charge at work	0.31**	0.04	[0.23, 0.38]	Supported
Hypothesis 2	Trust in supervisor mediates supervisor psychological closeness → Employee taking charge at work	0.05**	0.02	[0.02, 0.09]	Supported
Hypothesis 3a	Power distance orientation moderates supervisor psychological closeness → Employee taking charge at work	−0.09**	0.04	[−0.17, −0.02]	Supported
	Low (−1 SD)	0.35**	0.06	[0.24, 0.46]	
	Low (+1 SD)	0.15**	0.06	[0.03, 0.27]	
Hypothesis 3b	Moderated mediation: supervisor psychological closeness × Power distance orientation → Trust in supervisor → Employee taking charge at work	−0.02**	0.01	[−0.04, −0.01]	Supported
	Low (−1 SD)	0.07**	0.03	[0.02, 0.13]	
	High (+1 SD)	0.03**	0.02	[0.01, 0.08]	

**p ≤ 0.01.

**Figure 2:**
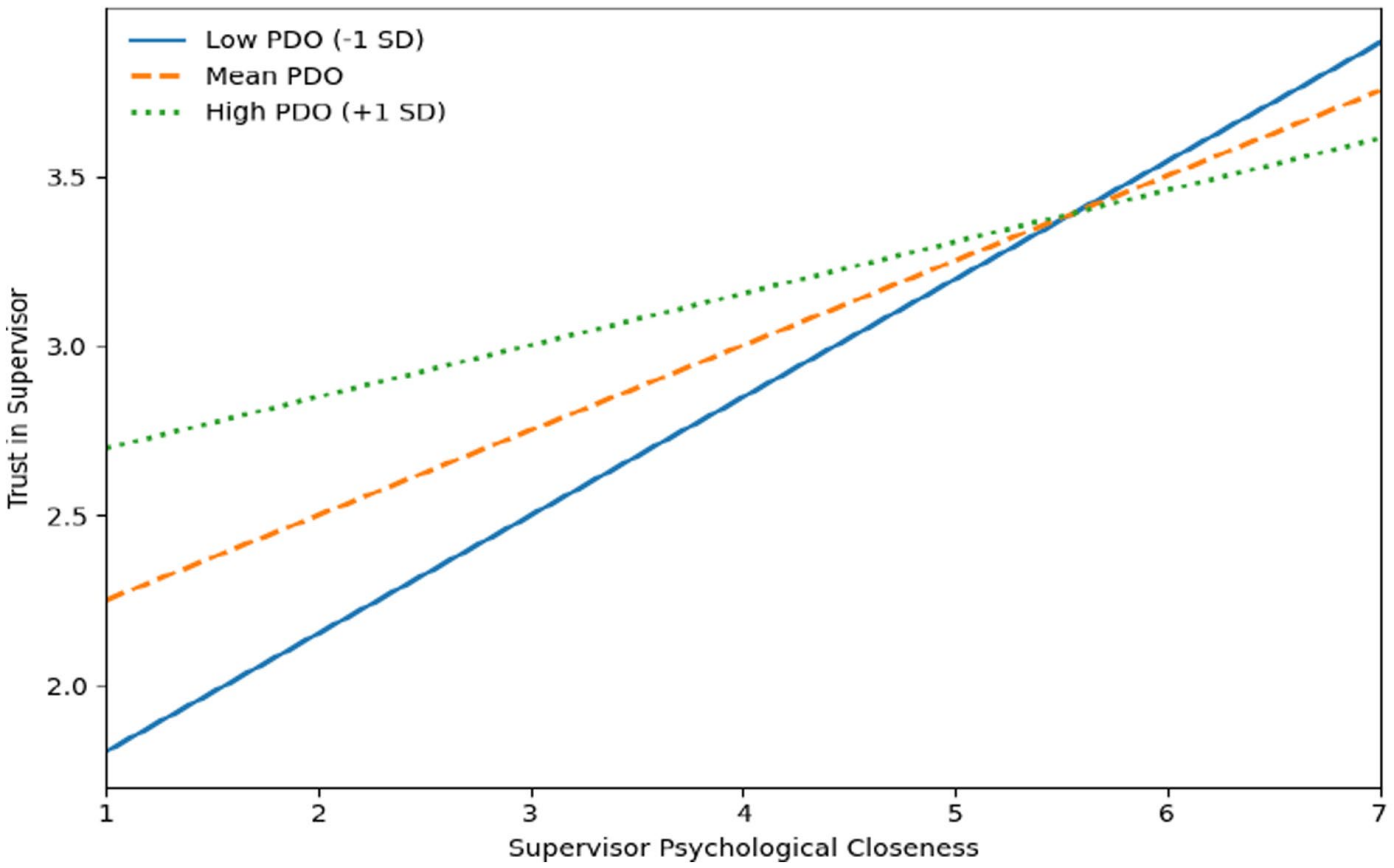
Moderating effect of power distance orientation.

Hypothesis 3a predicted that employee power distance orientation would moderate the relationship between supervisor psychological closeness and trust in supervisor, such that the relationship would be stronger for employees with lower power distance orientation. Consistent with this prediction, the interaction effect was significant and negative (*B* = −0.09**, SE = 0.04, 95% CI [−0.17, −0.02]), indicating that the positive association between supervisor psychological closeness and trust in supervisor weakened as power distance orientation increased. Simple slope analyses further showed that the association between supervisor psychological closeness and trust in supervisor was stronger at low power distance orientation (*B* = 0.35**, SE = 0.06, 95% CI [0.24, 0.46]) than at high power distance orientation (*B* = 0.15**, SE = 0.06, 95% CI [0.03, 0.27]), supporting Hypothesis 3a (see Figure 2).

Finally, Hypothesis 3b proposed a moderated mediation effect in which supervisor psychological closeness would influence employee taking charge at work through trust in supervisor, conditional on employee power distance orientation. The results supported this hypothesis, as the index of moderated mediation was significant (Index = −0.02**, BootSE = 0.01, 95% CI [−0.04, −0.01]). Conditional indirect effects further showed that the indirect effect of supervisor psychological closeness on employee taking charge at work via trust in supervisor was stronger at low levels of power distance orientation (*B* = 0.07**, BootSE = 0.03, 95% CI [0.02, 0.13]) than at high levels of power distance orientation (*B* = 0.03**, BootSE = 0.02, 95% CI [0.01, 0.08]). These results indicate that the mediated relationship was more pronounced when employee power distance orientation was low.

## Discussion

### Theoretical implications

Our study advances the literature on proactive work behavior in several important ways. First, we broaden the theoretical landscape of taking charge behavior by introducing supervisors’ psychological closeness as a pivotal, yet largely overlooked, relational antecedent. Whereas existing research has predominantly emphasized structural factors (e.g., job autonomy) or employee dispositional traits (e.g., proactive personality) as drivers of taking charge (Guzman et al., 2024; Kong and Lu, 2024), we pivot the conversation toward the relational fabric of the supervisor-employee dyad. By doing so, we respond to recent calls for a more relationally oriented understanding of proactivity (Xu et al., 2023; Zhang et al., 2024), demonstrating that affective proximity and supervisors’ emotional availability can act as vital psychological resources that energize employees to step beyond their formal roles. This shift underscores the importance of relational contexts in fostering agency and self-initiated workplace change.

Second, by conceptualizing and empirically validating trust in the supervisor as a mediating mechanism, we enrich the theoretical explanation of *how* and *why* psychological closeness translates into proactive behavior. Trust serves as a critical social-cognitive pathway (Zheng et al., 2019), providing employees with psychological safety, confidence, and a sense of support necessary to engage in risk-taking behaviors, such as taking charge. Through this mechanism, our study provides a more nuanced view of the relational processes that underpin employee proactivity, moving beyond surface-level associations to reveal the deeper psychological structures at play within high-quality supervisory relationships.

Third, we extend existing models by integrating employee power distance orientation as a moderating variable that conditions the effects of psychological closeness. By doing so, we address a gap in the literature concerning the boundary conditions of relational influences on proactive behavior. Our findings suggest that employees’ culturally rooted values regarding hierarchy and authority shape how relational resources, such as psychological closeness, are perceived and utilized. This contribution introduces critical cross-cultural sensitivity into proactive behavior research, emphasizing that relational interventions by managers may not be equally effective for all employees and are filtered through individual differences in power distance orientation. This insight adds complexity and cultural nuance to our understanding of when and for whom relational factors matter most.

### Practical implications

Our findings provide several actionable insights for organizations seeking to foster employee proactivity. First, managers should recognize the critical role of relational dynamics—specifically, psychological closeness—in encouraging employees to take charge. Organizations can train supervisors to develop emotional availability, genuine care, and closer interpersonal bonds with their employees, thereby fostering a relational environment that energizes initiative and proactive change behaviors. Second, by highlighting trust as a key mechanism, our study suggests that organizations should prioritize building and maintaining trust between supervisors and employees. Practical steps could include leadership development programs that emphasize trust-building behaviors such as consistent communication, transparency, fairness, and demonstrating concern for employee wellbeing. Supervisors who are perceived as trustworthy create psychological safety, empowering employees to challenge the status quo without fear of negative repercussions.

Third, recognizing the moderating role of employees’ power distance orientation implies that relational strategies should not be one-size-fits-all. Managers should be sensitive to individual differences in employees’ expectations regarding authority and hierarchy. For employees with a high power distance orientation, supervisors may need to strike a balance between closeness and demonstrating appropriate authority and respect for formal structures. Tailoring relational approaches to align with employees’ cultural values will help maximize the positive effects of psychological closeness on proactive behavior. Overall, organizations that foster strong, trust-based supervisory relationships while being mindful of individual and cultural differences will be better positioned to activate employees’ proactive potential and drive constructive organizational change.

### Limitations and future directions

Although our study employed a multi-source design, collecting data from both employees and supervisors across different organizations to enhance the robustness and generalizability of our findings, certain limitations still warrant consideration. First, while using multiple sources reduces concerns about common method bias, the correlational nature of the data still limits the ability to draw definitive causal conclusions. Future research could strengthen causal inferences by using experimental or longitudinal methods that capture changes in psychological closeness, trust, and taking charge behavior over time. Second, while we focused on supervisors’ psychological closeness as a key relational antecedent, other relational dynamics—such as leader humility, emotional support, or perceived supervisor fairness—may also play critical roles in fostering proactive behavior. Future studies could explore these additional relational factors, possibly in interaction with psychological closeness, to develop a more comprehensive understanding of the relational context that influences employee proactivity.

Third, the present study was conducted in China, a cultural context generally characterized by relatively high power distance. This context is particularly relevant to our model, as employees in high power distance settings may place greater importance on hierarchical relationships and may respond differently to supervisor closeness and trust than employees in more egalitarian cultures. Although our findings highlight the moderating role of individual power distance orientation, the broader cultural context may also shape how relational closeness translates into trust and taking charge at work. Accordingly, caution is warranted in generalizing these findings to cultures that differ substantially from China on this dimension. Future research should replicate this model in low power distance contexts to examine whether the observed relationships remain similar in strength and direction across cultures. Such work would offer a more culturally grounded understanding of how supervisor–employee relational dynamics influence taking charge behavior. Finally, although our focal outcome was taking charge at work, supervisor psychological closeness and trust in supervisor may also affect other important work outcomes, such as innovation, employee voice, job crafting, and readiness for change. Future research could examine these outcomes to develop a more comprehensive understanding of the role of relational closeness in dynamic organizational settings.

## Conclusion

Grounding on social exchange theory, the study results show that supervisors’ psychological closeness is positively linked to employees taking charge at work. Moreover, trust in the supervisor mediates the path between supervisors’ psychological closeness and employee taking charge at work. Additionally, employee power distance orientation moderates the path between supervisors’ psychological closeness and trust in the supervisor, directly and indirectly strengthening the mediated relationship. These findings underscore the importance of managers recognizing the interplay between relational and cultural influences to promote proactive behaviors among employees effectively.

## Data Availability

The raw data supporting the conclusions of this article will be made available by the authors, without undue reservation.

## Appendix

### Supervisor psychological closeness

Please circle the picture below that best describes your relationship.
